# A deep convolutional neural network for efficient microglia detection

**DOI:** 10.1038/s41598-023-37963-8

**Published:** 2023-07-10

**Authors:** Ilida Suleymanova, Dmitrii Bychkov, Jaakko Kopra

**Affiliations:** 1 Faculty of Biological and Environmental Sciences, Helsinki Institute of Life Science (HiLIFE), University of Helsinki, Helsinki, Finland; 2 Institute for Molecular Medicine Finland (FIMM), Helsinki Institute for Life Science (HiLIFE), University of Helsinki, Helsinki, Finland; 3 Division of Pharmacology and Pharmacotherapy, Faculty of Pharmacy, University of Helsinki, Helsinki, Finland

**Keywords:** Image processing, Machine learning

## Abstract

Microglial cells are a type of glial cells that make up 10–15% of all brain cells, and they play a significant role in neurodegenerative disorders and cardiovascular diseases. Despite their vital role in these diseases, developing fully automated microglia counting methods from immunohistological images is challenging. Current image analysis methods are inefficient and lack accuracy in detecting microglia due to their morphological heterogeneity. This study presents development and validation of a fully automated and efficient microglia detection method using the YOLOv3 deep learning-based algorithm. We applied this method to analyse the number of microglia in different spinal cord and brain regions of rats exposed to opioid-induced hyperalgesia/tolerance. Our numerical tests showed that the proposed method outperforms existing computational and manual methods with high accuracy, achieving 94% precision, 91% recall, and 92% F1-score. Furthermore, our tool is freely available and adds value to exploring different disease models. Our findings demonstrate the effectiveness and efficiency of our new tool in automated microglia detection, providing a valuable asset for researchers in neuroscience.

## Introduction

Microglial cells are immune cells of the central nervous system (CNS), representing 10–14% of all glia^1^. Different studies report the activation of microglia in glaucoma^2^, neuropathic pain^3^, viral^4^, bacterial^4^ and parasitic^4^ infections. They are also essential to learning and memory^5,6^ and protect neurons from damage. Microglia spread inflammatory signals in response to even small pathological changes in the CNS^7^.

Microglial cells have small, rounded bodies with large and ramified branches. These cells spread throughout the nerve tissue but without overlapping adjacent cells. Developing fully automated methods for counting microglia cells from immunohistological images with no user-defined parameters is a significant challenge in the field. Traditional CNS microglia quantification techniques are manual or semi-automated. Manual counting is time-intensive and involves human error. Several detection approaches have been developed for fluorescently stained microglia^8–11^. For example, Kozlowski et al.^9^ used the Outsu threshold method, and de Gracia et al.^8^ fixed the manual threshold. Detecting the positive cells in a fluorescent image is very simple because the positive signal of the cells is substantially higher than the background^12^. Microglia markers can also be visualised using a more sensitive version of immunohistochemistry: secondary antibodies conjugated with 3,3′-diaminobenzidine (DAB). DAB staining is typically more sensitive and typically enables measuring the more detailed texture of the cells^13^. DAB staining is known for making the definition of a positive object vast and is, therefore, more challenging to quantify than the fluorescence-stained cells^14^. One possible solution is using or developing ImageJ plugins^15,16^. Morrison et al. applied ImageJ plugins to segment DAB-stained microglia^16^. To achieve this goal, they utilised skeletal and fractal analyses while manually counting the number of cells. The use of plugins, however, presents a significant limitation due to their inconsistent and often unpredictable performance, which is influenced by various factors. Moreover, the method is not entirely automated.

Deep convolutional neural networks (DCNN)-based models are a way to overcome many shortcomings of manual or semi-automated methods in cell detection^17^. DCNN applications outperformed traditional methods in 2012, attracting increasing attention to computational cell biology and healthcare^18–27^. DCNN models are successful, notably in complex cell classification tasks^28^. Kyriazis et al. proposed a custom DCNN model, but this method is inaccurate^29^. In the recent work, Stetzik et al. trained the DCNN model as part of the commercial software Aiforia™ to detect DAB-stained microglia in mouse models of viral infection^30^. The main limitations of commercial software are that they are costly, and the performance is time-consuming. Furthermore, there are no universally best tools, and many of these tools require manual changes, which strongly limits their efficient use within the biologist community^31–33^.

Several methods have been developed to detect microglia in past years using traditional image processing tools and DCNN^8–11,15,16,29,30^. Our research aim was to develop a fully automated tool for microglia detection that would be more accurate, efficient, and faster than existing approaches. We present an innovative algorithm for the automatic detection of microglia based on YOLOv3^34^—a powerful DCNN platform that can be customised to deal with a range of object detection tasks (Supplementary Information). A significant advantage of this platform is that it can be tuned to deal with a range of object detection tasks. This platform enables a simple selection of the deep learning architecture size, which can be matched to the object detection task's complexity, allowing the network to be trained with a relatively small number of annotated images. Furthermore, this platform delivers competitive performance without requiring extensive training processes or optimisation of various hyperparameters.

In this project, we used sections from the control and opioid-induced hyperalgesia/tolerance (OIH/OIT) groups described by Jokinen et al.^35^. OIH/OIT refers to the dose escalation during long-term opioid therapy, which can lead to increased pain^36^. Research has shown that the long-term use of morphine can cause glia activation^37^. This activation can lead to the production and release of various neuro-excitatory substances^38,39^. Microglia tend to undergo extensive morphological changes during activation, significantly increasing variability in size and shape and showing complex arrangements of their processes and networks^36^. For our analysis, we chose specific brain and spinal cord regions believed to be involved in chronic pain management^35^.

To demonstrate the effectiveness of our microglia detection, we trained YOLOv3 using its general network architecture. To validate the performance of YOLOv3, we compared it with other commonly used approaches such as expert observer's detection, ImageJ, and semi-automated tools such as *ilastik*. In addition, we evaluated the detection accuracy in an experimental biological task by counting the number of cells in the forelimb motor cortex under the effect of morphine. We selected the forelimb motor cortex brain sections as we had previously reported changes in the number of microglia in this area^35^. Our results show that our algorithm performs exceptionally well compared to modern methods in terms of accuracy and computational efficiency.

## Materials and methods

### Animals

The study protocol was approved by the experimental animal ethics committee of the provincial government of Southern Finland (Uudenmaan Lääninhallitus, Hämeenlinna, Finland, permission # ESAVI/7929/04.10.07/2014). All methods were performed in accordance with the relevant guidelines and regulations of the International Association for the Study of Pain^40^, and European Communities Council Directive, 24 November 1986. All experiments were performed in accordance with ARRIVE guidelines. Ten adult male Sprague–Dawley rats (from Scanbur, Sollentuna, Sweden) weighing 225 ± 25 g (mean ± SEM) were used at the beginning of experiment^35^. Rats were divided into an OIH/OIT group and a control group (saline), with five rats per each group^35^. The animal model, including precise biological experiments, is published by Jokinen et al.^35^.

### Preparation of samples

The experimental dataset utilised different regions of the rat brain to examine the efficiency of the proposed approach. Tissue sections of 6 μm were prepared from selected regions of the brain and lumbar regions of the spinal cords as previously described^35^. The selected sections were labelled with antibody for Ionized calcium-binding adapter molecule 1 (Iba1) (1:1000, Catalogue No. 019-19741, Wako, Richmond, VA, USA), using anti-rabbit and anti-mouse biotinylated secondary antibodies and the VECTASTAIN ABC HRP Kit (Cat PK-6101, PK-4002 Vector Laboratories, Burlingame, CA, USA)^35^. Slides were scanned and imaged by the 3DHISTECH Scanner (3DHISTECH Ltd, Budapest, Hungary).

### Annotation procedure

Annotations were made manually using a graphical image annotation tool LabelImg. 22 images and 6500 cells were labelled for the training set. All images and data analysed during the study are included in Supplementary Information. The cells were inserted into the training set by an expert drawing a bounding box around them. The training data was annotated twice by the expert. In the second round of cell labelling, all images were rotated 180° to evaluate intra-person accuracy.

### Convolutional neural network

Using general default architecture of YOLOv3, as a one-step object detection algorithm, YOLOv3 transforms the detection problem into a regression problem^34,41,42^. YOLOv3 uses darknet-53 as a backbone and binary cross-entropy loss function. The Darknet53 network consists of a convolutional layer and a residual block.

The residual network employed by Darknet53 can be represented:

$$\begin{aligned} X_{{1}} = & \sigma \{ \beta (W_{{1}} ,X)\} \\ X_{{2}} = & \sigma \{ \beta (W_{{2}} ,X_{{1}} )\} \\ X_{{3}} = & X + X_{{{2} }} \\ \end{aligned}$$

where *X* is an input feature, (*W*_1_, *X*) is an input feature, a weight of *W*_1_ is a weight, the size of the convolution kernel of *W*_1_ is 1 × 1. β is batch normalisation, σ is nonlinear ReLU activation. *X*_2_ is a backbone output feature of the residual structure, where the size of the convolution kernel of *W*_2_ is 3 × 3, *X*_3_ is a final output feature of the residual network. First, a traditional 3 × 3 convolution on the input features is performed, next step is stacks of five residual blocks. The residual network number of each residual block is 1, 2, 8, 8, and 4. Residual blocks are connected by the downsampling convolution.

During training, all hyperparameters were set: learning rate is 0.001; momentum is 0.9; weight decay is 0.0005; batch size is 32.

### Evaluation methods and metrics

We evaluated the detection results by calculating precision, recall and F1-score. A correct detection (true positive, TP) happens when a ground truth object has a matched pair, and false positive (FP) detection happens when an extra object is present. In contrast, a false negative (FN) happens in the case of missing objects. Based on these definitions we calculated precision [defined as P = TP/(TP + FP)], recall [defined as R = TP/(TP + FN)], and F1-score [defined as F1 = TP/(TP + (FP + FN) × 0.5)].

The same example images of cells were mirrored and rotated, and blindly shown to the annotators to be labelled again to reduce bias to the lowest possible level. Altogether the annotators manually annotated ~ 10 different images containing ~ 650 cells using the LabelImg tool.

In *ilastik*, we used pixel and object classifications with Gaussian Smoothing colour/intensity feature, Gaussian Gradient Magnitude and Difference of Gaussian edge features. In ImageJ, we used global threshold maximum entropy since it performed best to other available global and local thresholds in ImageJ. All pixels outside the threshold were set to zero. Small objects were deleted as noise objects based on the object size threshold.

Correlation analysis was performed with the Pearson correlation test. The statistical test was performed in Matlab.

## Results

**Figure 1:**
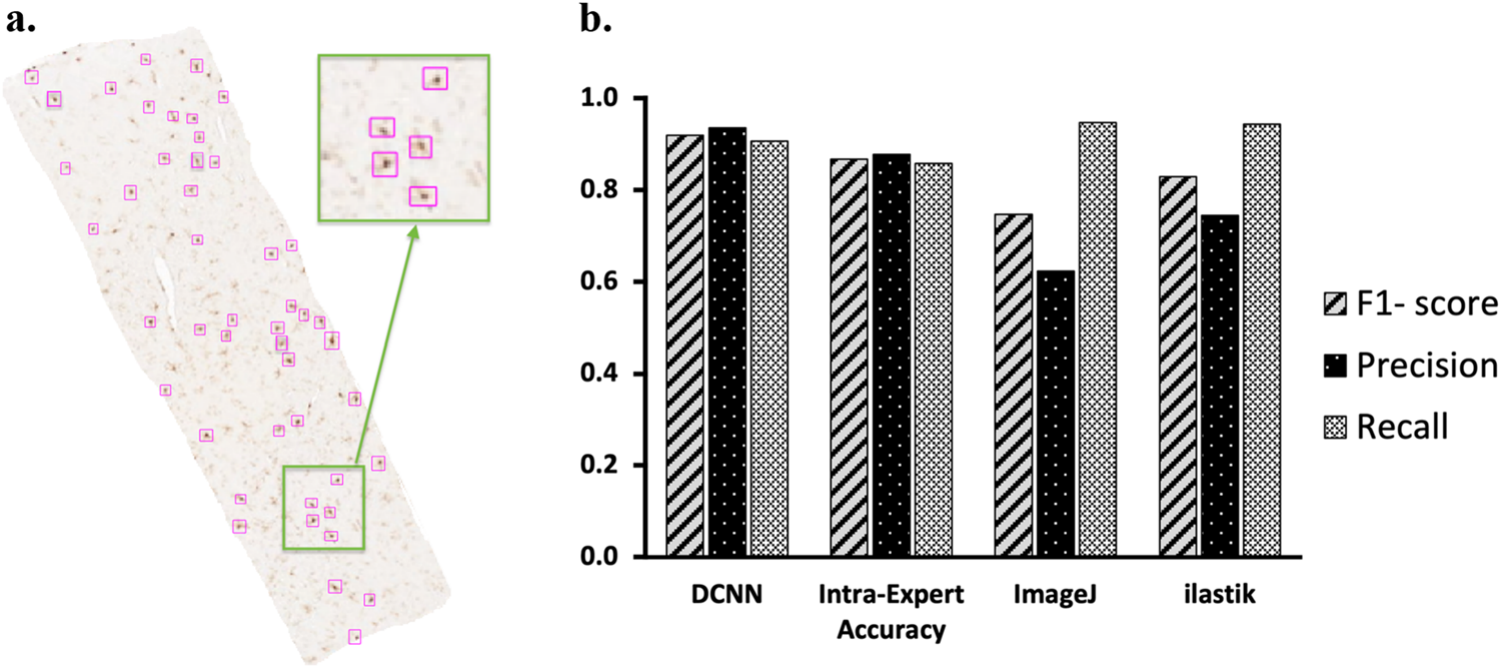
Examples of microglia detection by FindMyCells. (**a**) Detection results. (**b**) Evaluated metrics for DCNN, manual counting, ImageJ, and *ilastik*.

Using precision, recall, and F1-score metrics, we evaluated the detection results from DCNN by comparing them with manual counting, *ilastik*, and ImageJ. Figure 1a presents examples of detected microglia by DCNN.

As shown in Fig. 1b, DCNN achieved precision, recall, and F-1 score values P = 0.94, R = 0.91, and F1 = 0.92, respectively. Whereas precision and recall values for *ilastik* were P = 0.74, R = 0.94, F1 = 0.83, and for ImageJ P = 0.62, R = 0.95, F1 = 0.75, for manual counting P = 0.88, R = 0.86, F1 = 0.87. As expected, our method generally performed very competitively. We hypothesise that possible reasons for any discrepancy in the accuracy are related to the atypical shape of microglia or/and variability in staining intensity. Statistics for all evaluated metrics are shown in the Supplementary Table.

In practice, the runtime of a method is also an important factor. DCNN is approximately 170 times faster than manual counting, 60 times less than *ilastik*, and 30 times less than ImageJ. This indicates that the DCNN model is characterised by a highly beneficial cell detection time besides its good quality performance. Annotation of 6500 cells took ~ 4 h of continuous labelling, and for training, the model took ~ 45 h of training time, 300 epochs. The annotated cells were chosen to include the training data from various quality images, representative variations in staining, tissues preparations, and imaging (Supplementary Information). Taken together, DCNN has appears to have a very favourable runtime, in addition to good performance.

**Figure 2:**
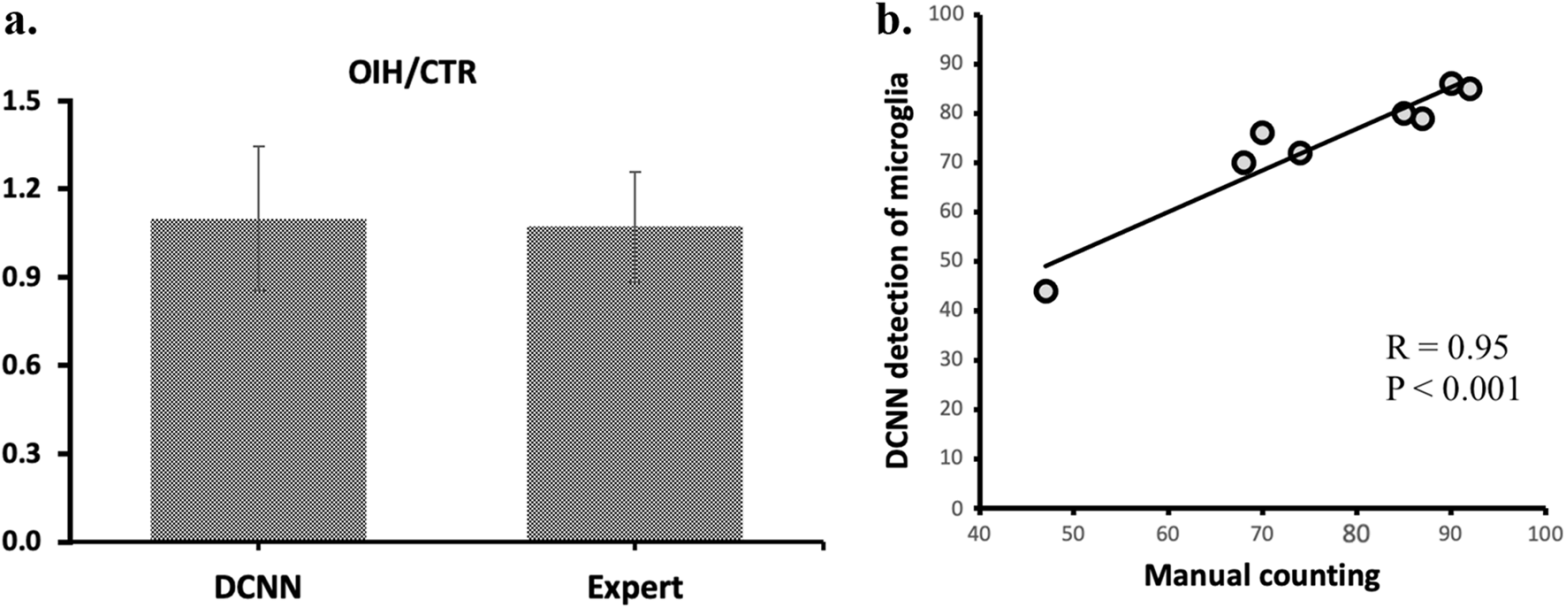
Microglia detection in OIH/OIT and CTR groups. (**a**) Detection results ratio OIH group to CTR group. (**b**) Spearman correlation between the DCNN model and manual counting.

Additionally, we applied the proposed model for detecting cells in forelimb motor cortex brain sections of CTR and OIH/OIT groups. The microglia ratio between groups is 1.12 for manual counting and 1.13 for DCNN, indicating only ~ 2% measurement difference (Fig. 2a). The newly developed model is highly correlated with the manual counting method, with Spearman correlation of R = 0.95 (Fig. 2b).

## Discussion and conclusions

Our approach introduces a novel, fully automated method for accurately detecting microglia, which can aid to better understand their pathophysiological processes.

Unlike traditional object detection models that use multiple stages, YOLOv3 uses a single neural network^43,44^. YOLOv3 can achieve high accuracy in object detection while remaining fast due to its functional pyramidal network and prediction engine. In addition, it offers excellent speed for detecting objects of different sizes, in a wide range of settings and scenarios, which is crucial in cell detection. General architecture YOLOv3 was chosen to perform microglia detection since it has been designed to be fast and efficient^34^, making it rather ideal for the task. 6500 cells were manually labelled to train the model. To benchmark our detection approach, we compared it against ImageJ, machine learning-based tool *ilastik*, and manual detection. The model achieved F1 score of 92% and significantly outperformed other approaches. In addition, DCNN output showed high correlation compared to the manual microglia counting, which demonstrates its validity. Notably, the F1 score of the human expert was 4% units lower than YOLOv3 (88% vs 92%). We consider that the intra-observer accuracy indicates the complex nature of microglia.

We also want to remark that DAB-based staining of cells results in a significantly wider staining intensity between the samples than fluorescent dyes. Intensity inhomogeneity is a significant issue in the analysis of medical images and can greatly undermine the performance of image analysis processing and segmentation^45^. Several approaches are generally used to overcome this issue from magnetic resonance images^46,47^. In this work, the high performance of the proposed approach has been obtained without any intensity normalisation.

Kyriazis et al. and Stetzik et al. also used DCNN model to detect microglia^29,30^. However, Kyriazis's method could correctly recognise only 70% of cells. The authors used only 300 cells for training the DCNN model. The authors expected to improve performance with higher volume of training data. Stetzik et al. implemented the user-friendly commercial software Aiforia™, where the main drawback is that the computational time for cell recognition is hundred times slower compared to ours. In addition, the Aiforia™ platform is also costly, limiting its scalability in an academic setting.

Our research has some limitations that require addressing in future studies. Firstly, all materials were obtained from rat opioid models, so the validity and applicability of the proposed method in other organisms and models should be confirmed. Additionally, the default deep convolutional neural network architecture was utilised in the project, and further optimisation of the hyperparameters and increasing the number of annotated examples should further improve the model's performance. YOLOv3, as a larger network, requires high-performance hardware for excellent performance and has a fixed input image size. Compared to region-based convolutional neural networks, the algorithm has a poorer ability to recognize object positions and a lower recall rate^48^. However, YOLOv3 performs better in detecting complex samples^49^.

In conclusion, our novel approach for microglia detection has demonstrated high performance in detecting microglial cells with significant variation in appearance, size, and shape. Our experiments have shown that our method outperforms several widely used approaches. The automated detection process provides a quick and reliable quantification of microglia, without the need for any user-defined parameters. Moving forward, we plan to further train our model to recognise fluorescently and DAB-stained cells and extract morphological features of microglia to gain insights into their precise mechanisms and regulatory functions. This tool has the potential to significantly advance our understanding of microglia and their role in various neurological conditions.

### Supplementary Information

Supplementary Information.

Supplementary Tables.

## Data Availability

All data used and analysed during this study are included in this published article (Supplementary Information).
