# Author Correction: A deep convolutional neural network for efficient microglia detection

**DOI:** 10.1038/s41598-024-51952-5

**Published:** 2024-01-18

**Authors:** Ilida Suleymanova, Dmitrii Bychkov, Jaakko Kopra

**Affiliations:** 1 Faculty of Biological and Environmental Sciences, Helsinki Institute of Life Science (HiLIFE), University of Helsinki, Helsinki, Finland; 2 Institute for Molecular Medicine Finland (FIMM), Helsinki Institute for Life Science (HiLIFE), University of Helsinki, Helsinki, Finland; 3 Division of Pharmacology and Pharmacotherapy, Faculty of Pharmacy, University of Helsinki, Helsinki, Finland

Correction to: *Scientific Reports* 10.1038/s41598-023-37963-8, published online 10 July 2023

In the Materials and methods sections, under the subheading ‘Preparation of samples’,

“The experimental dataset utilised different regions of the rat brain to examine the efficiency of the proposed approach. Tissue sections of 6 μm were prepared from selected regions of the brain and lumbar regions of the spinal cords as previously described^35^. The selected sections were labelled with antibody for GFAP (1:400, Cat G-3893, Sigma-Aldrich, St. Louis, MO, USA), using anti-rabbit and anti-mouse biotinylated secondary antibodies and the VECTASTAIN ABC HRP Kit (Cat PK-6101, PK-4002 Vector Laboratories, Burlingame, CA, USA)^35^. Slides were scanned and imaged by the 3DHISTECH Scanner (3DHISTECH Ltd, Budapest, Hungary).”

now reads:

“The experimental dataset utilised different regions of the rat brain to examine the efficiency of the proposed approach. Tissue sections of 6 μm were prepared from selected regions of the brain and lumbar regions of the spinal cords as previously described^35^. The selected sections were labelled with antibody for Ionized calcium-binding adapter molecule 1 (Iba1) (1:1000, Catalogue No. 019-19741, Wako, Richmond, VA, USA), using anti-rabbit and anti-mouse biotinylated secondary antibodies and the VECTASTAIN ABC HRP Kit (Cat PK-6101, PK-4002 Vector Laboratories, Burlingame, CA, USA)^35^. Slides were scanned and imaged by the 3DHISTECH Scanner (3DHISTECH Ltd, Budapest, Hungary).”

The original Article has been corrected.

