# Supplementary figures and images for: A deep convolutional neural network for efficient microglia detection
Source: Sci Rep. 2023 Jul 10;13:11139. doi: 10.1038/s41598-023-37963-8 (PMC10333175; doi:10.1038/s41598-023-37963-8)

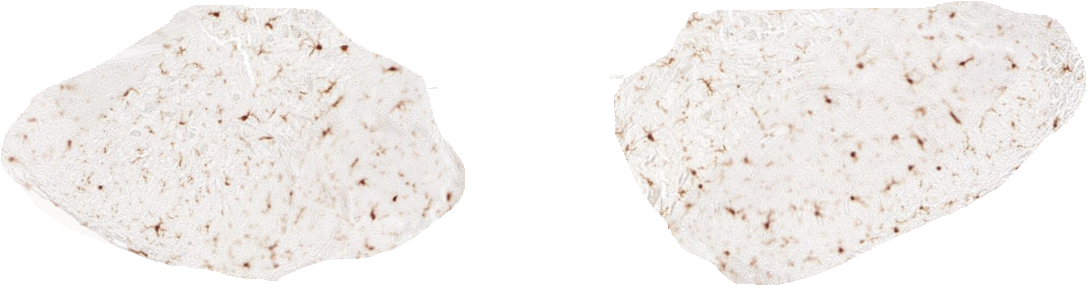

Supplement: Supplementary file 2 — Supplementary Tables. [file 41598_2023_37963_MOESM2_ESM.zip › 1-1M10-2_dorsal_horn.png]

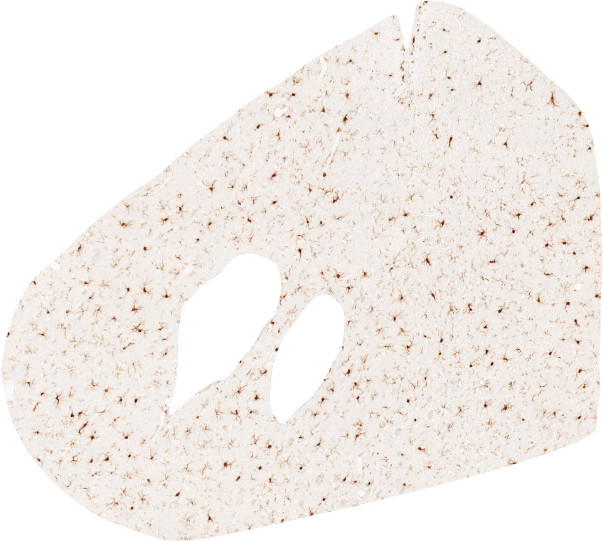

Supplement: Supplementary file 2 — Supplementary Tables. [file 41598_2023_37963_MOESM2_ESM.zip › 1M01-3acbc.png]

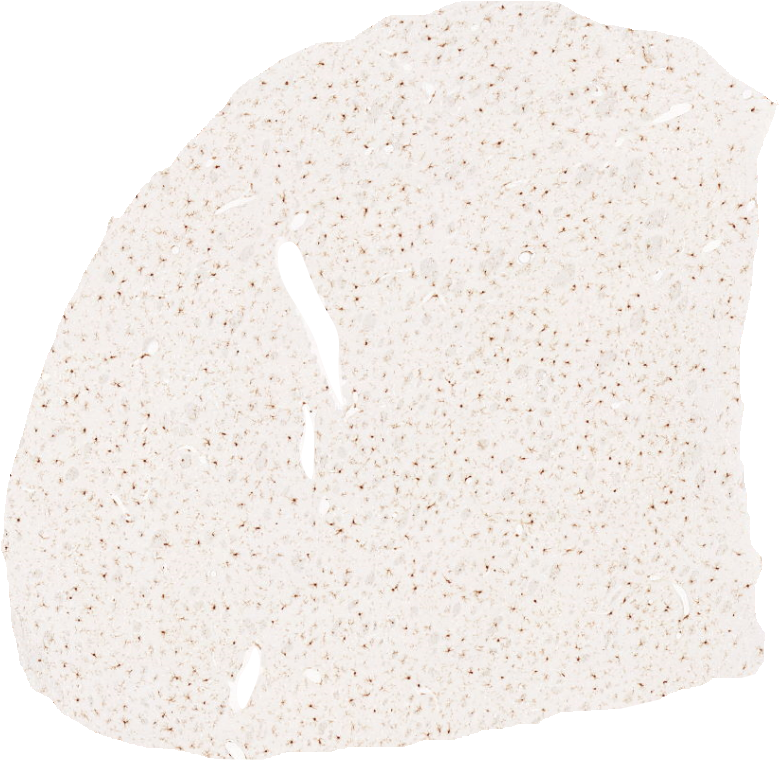

Supplement: Supplementary file 2 — Supplementary Tables. [file 41598_2023_37963_MOESM2_ESM.zip › 1M01-4_Str.png]

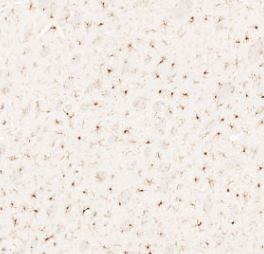

Supplement: Supplementary file 2 — Supplementary Tables. [file 41598_2023_37963_MOESM2_ESM.zip › 1M01-4_Striatum_cropped.png]

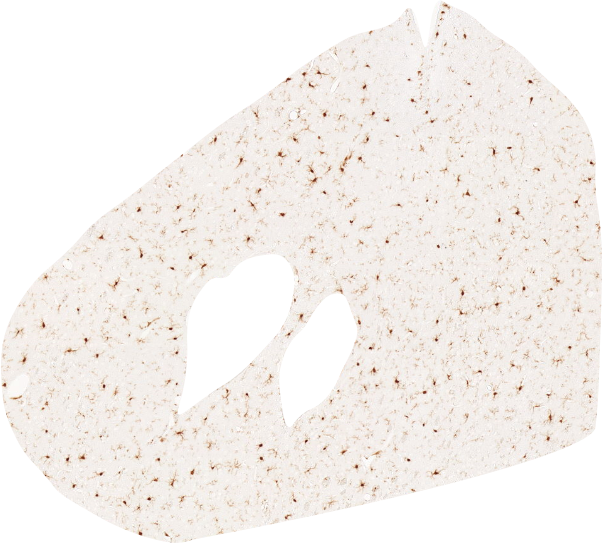

Supplement: Supplementary file 2 — Supplementary Tables. [file 41598_2023_37963_MOESM2_ESM.zip › 1M01-6acbc_test_image.png]

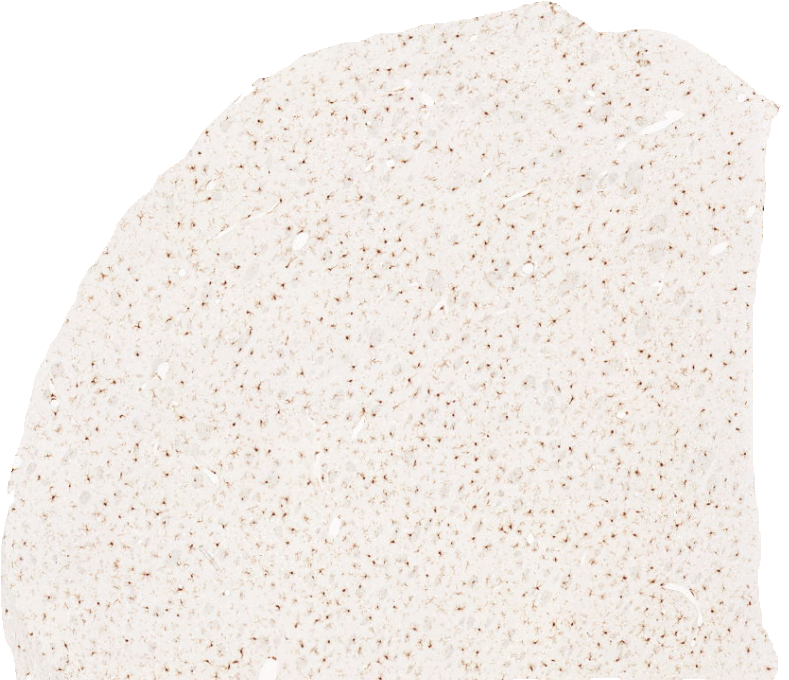

Supplement: Supplementary file 2 — Supplementary Tables. [file 41598_2023_37963_MOESM2_ESM.zip › 1M01_Str.png]

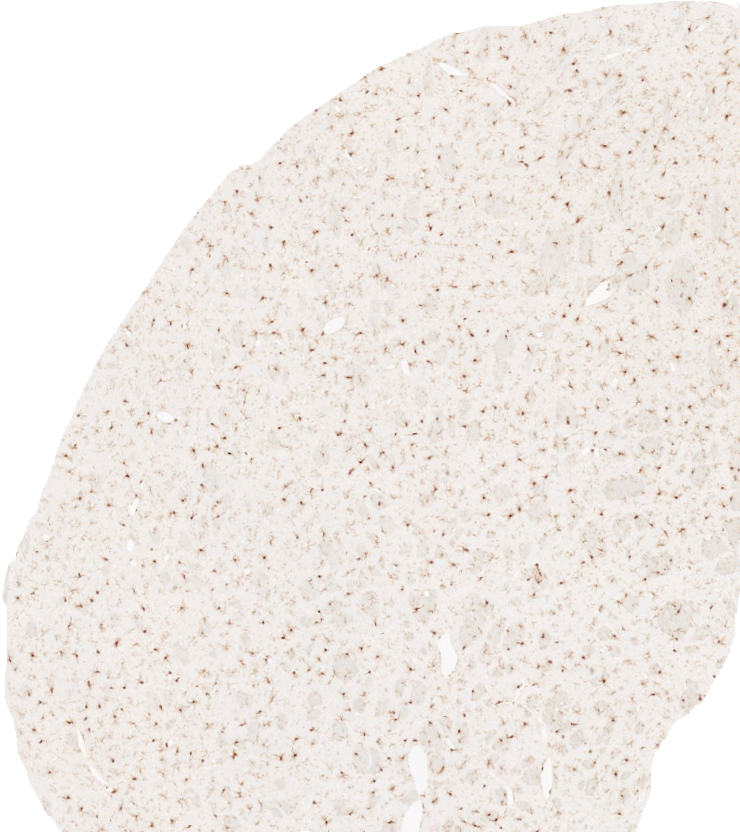

Supplement: Supplementary file 2 — Supplementary Tables. [file 41598_2023_37963_MOESM2_ESM.zip › 1M05-3_Str.png]

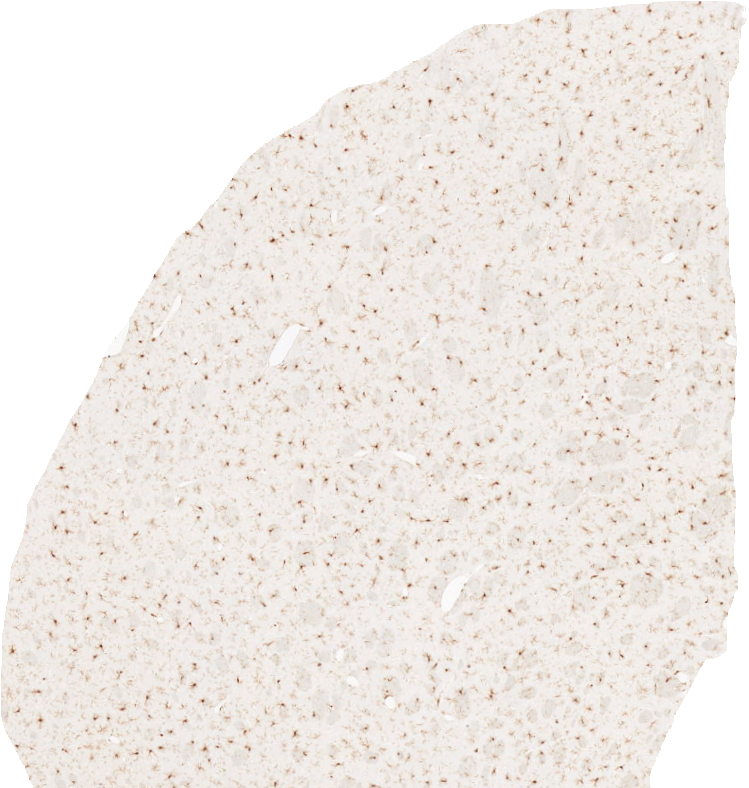

Supplement: Supplementary file 2 — Supplementary Tables. [file 41598_2023_37963_MOESM2_ESM.zip › 1M05_Str.png]

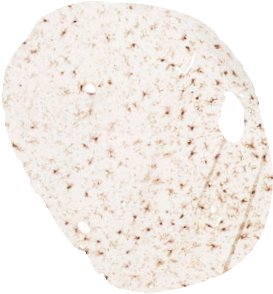

Supplement: Supplementary file 2 — Supplementary Tables. [file 41598_2023_37963_MOESM2_ESM.zip › 1M06-2_Central_amygdaloid.png]

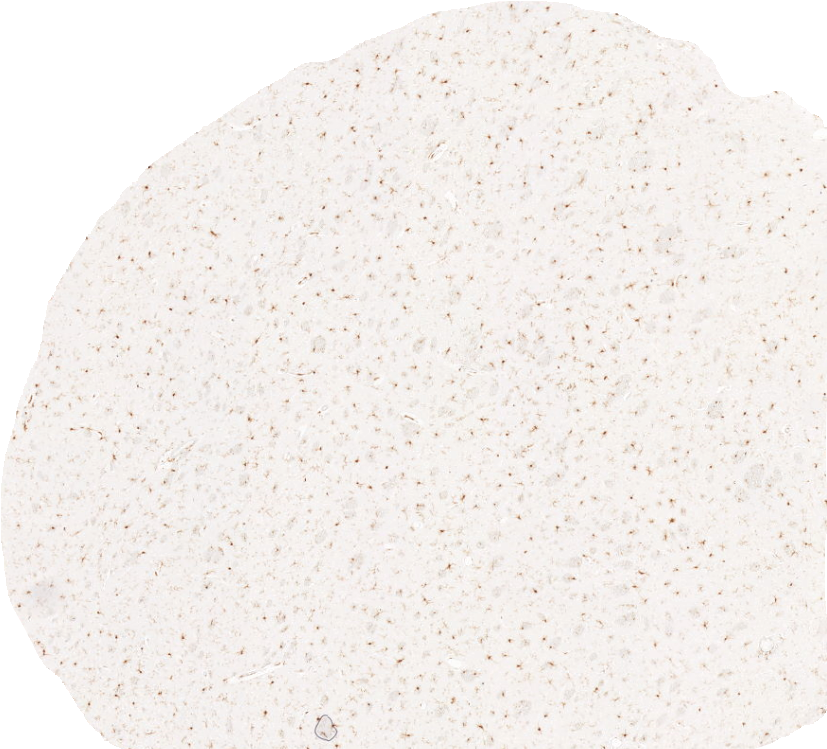

Supplement: Supplementary file 2 — Supplementary Tables. [file 41598_2023_37963_MOESM2_ESM.zip › 1M12-4_Str.png]

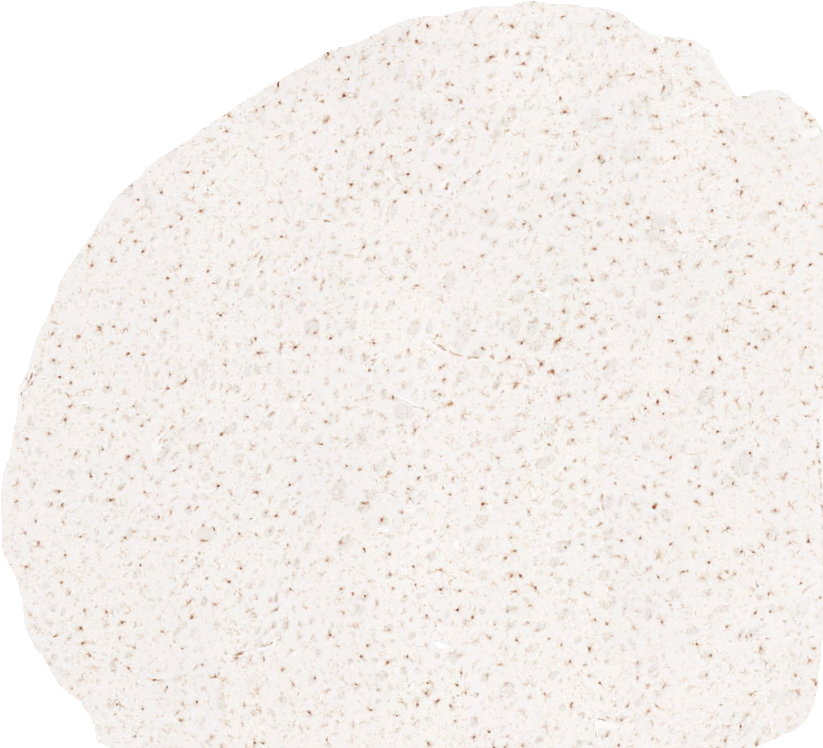

Supplement: Supplementary file 2 — Supplementary Tables. [file 41598_2023_37963_MOESM2_ESM.zip › 1M12_Str.png]

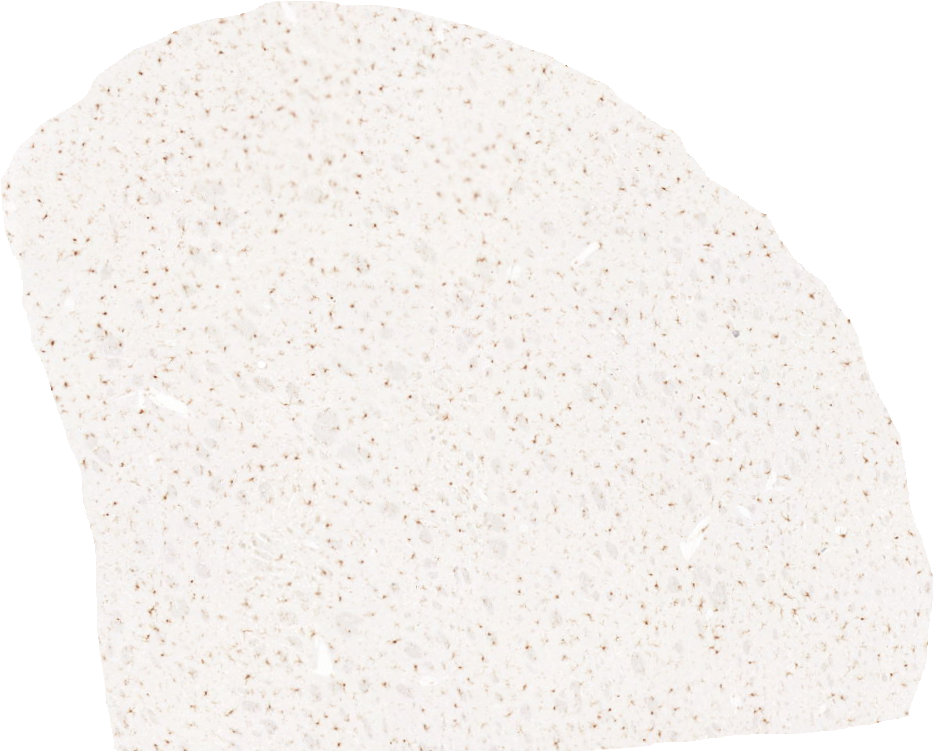

Supplement: Supplementary file 2 — Supplementary Tables. [file 41598_2023_37963_MOESM2_ESM.zip › 1M16-4_Str.png]

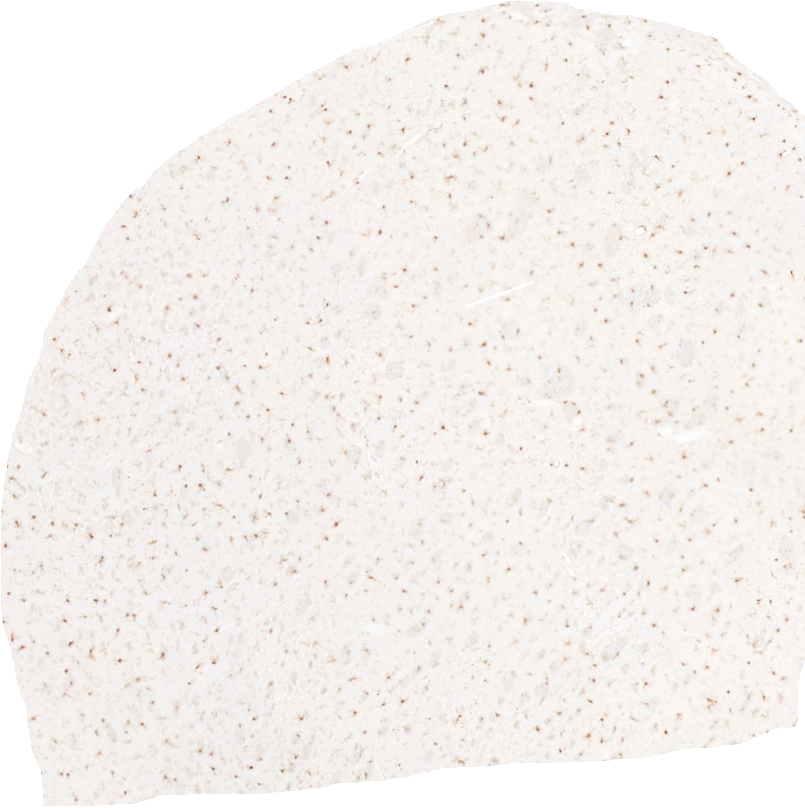

Supplement: Supplementary file 2 — Supplementary Tables. [file 41598_2023_37963_MOESM2_ESM.zip › 1M16_Str.png]

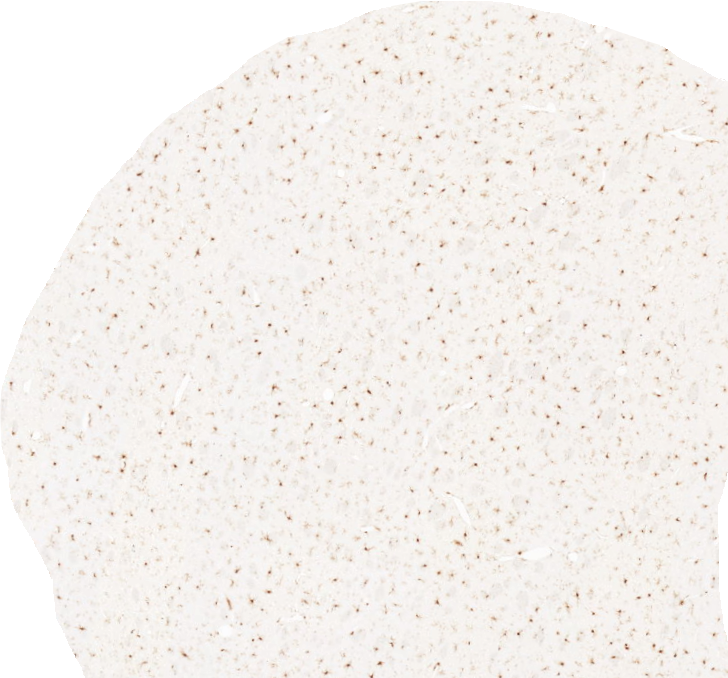

Supplement: Supplementary file 2 — Supplementary Tables. [file 41598_2023_37963_MOESM2_ESM.zip › 1M20-4_Str.png]

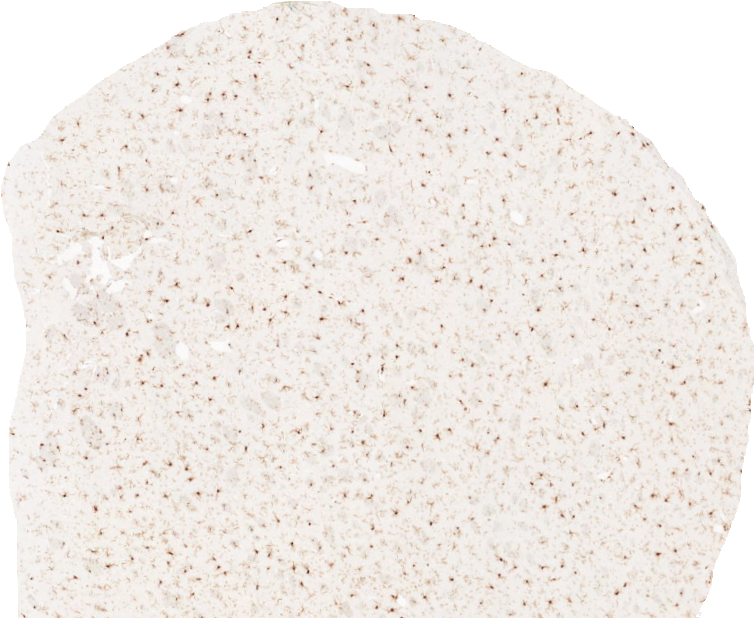

Supplement: Supplementary file 2 — Supplementary Tables. [file 41598_2023_37963_MOESM2_ESM.zip › 1M24_Str.png]
